# Small molecules disaggregate alpha-synuclein and prevent seeding from patient brain-derived fibrils

**DOI:** 10.1073/pnas.2217835120

**Published:** 2023-02-09

**Authors:** Kevin A. Murray, Carolyn J. Hu, Hope Pan, Jiahui Lu, Romany Abskharon, Jeannette T. Bowler, Gregory M. Rosenberg, Christopher K. Williams, Gazmend Elezi, Melinda Balbirnie, Kym F. Faull, Harry V. Vinters, Paul M. Seidler, David S. Eisenberg

**Affiliations:** ^a^Departments of Chemistry and Biochemistry and Biological Chemistry, UCLA-DOE Institute, Molecular Biology Institute, UCLA, Los Angeles, CA 90095; ^b^HHMI, UCLA, Los Angeles, CA 90095; ^c^Department of Pathology and Laboratory Medicine, David Geffen School of Medicine, UCLA, Los Angeles, CA 90095; ^d^Pasarow Mass Spectrometry Laboratory, David Geffen School of Medicine, UCLA, Los Angeles, CA 90095; ^e^Department of Neurology, David Geffen School of Medicine, UCLA, Los Angeles, CA 90095; ^f^Department of Pharmacology and Pharmaceutical Sciences, University of Southern California, Los Angeles, CA 90089

**Keywords:** Parkinson's, amyloid, disaggregation, multiple system atrophy

## Abstract

The amyloid aggregation of alpha-synuclein within the brain is associated with the pathogenesis of Parkinson’s disease (PD) and other related synucleinopathies, including multiple system atrophy (MSA). Alpha-synuclein aggregates are a major therapeutic target for treatment of these diseases. We identify two small molecules capable of disassembling preformed alpha-synuclein fibrils. The compounds, termed CNS-11 and CNS-11g, disaggregate recombinant alpha-synuclein fibrils in vitro, prevent the intracellular seeded aggregation of alpha-synuclein fibrils, and mitigate alpha-synuclein fibril cytotoxicity in neuronal cells. Furthermore, we demonstrate that both compounds disassemble fibrils extracted from MSA patient brains and prevent their intracellular seeding. They also reduce in vivo alpha-synuclein aggregates in *C. elegans*. Both compounds also penetrate brain tissue in mice. A molecular dynamics–based computational model suggests the compounds may exert their disaggregating effects on the N terminus of the fibril core. These compounds appear to be promising therapeutic leads for targeting alpha-synuclein for the treatment of synucleinopathies.

## Significance

Parkinson’s disease (PD) and the related condition multiple system atrophy (MSA) are associated with the aggregation of the protein alpha-synuclein into fibrils within the brain. Disaggregation of these fibrils could possibly slow or reverse the progress of these diseases. As a step toward testing this hypothesis, we have identified two small molecules that disassemble preformed fibrils of alpha-synuclein in the test tube, including fibrils from postmortem patient brains. These two compounds penetrate the mouse brain and reduce aggregation of alpha-synuclein in the worm *C. elegans*; thus, they may merit development into therapeutics for PD and MSA.

Parkinson’s disease (PD), multiple system atrophy (MSA), and dementia with Lewy bodies (DLB) are neurodegenerative disorders characterized by abnormal accumulation of the protein alpha-synuclein (1). Known as synucleinopathies, these diseases are hallmarked by the fibrillar aggregation of alpha-synuclein in either neurons or glial cells (2). Alpha-synuclein aggregation is potentially causative of disease progression as variants in alpha-synuclein that promote aggregation are associated with early-age disease onset and familial forms of PD and DLB (3). In PD, alpha-synuclein aggregation occurs primarily in dopaminergic neurons, while in MSA, aggregation is primarily in oligodendrocytes (4, 5). Natively, alpha-synuclein functions as a vesicle transport protein and more recently found to be involved in P-body and mRNA stability (6, 7). Alpha-synuclein is an intrinsically disordered protein, which has made the determination of an atomic structure of its soluble form challenging. It has been shown that soluble alpha-synuclein adopts a helical form when bound to cell membranes, while its aggregated form adopts the cross-beta structure typical of other amyloid fibrils (8–10). Multiple structures of fibrillar alpha-synuclein have been determined both of recombinant (11–16) and brain-derived fibrils (17, 18).

In addition to primary aggregation, one mechanism by which alpha-synuclein pathology spreads throughout the brain is the prion-like seeding of alpha-synuclein aggregates (19). Alpha-synuclein fibrils can seed the aggregation of soluble native protein, and Lewy body pathology is observed to spread through connected brain regions (19). Aggregation of alpha-synuclein, both primary and seeded, has become a main therapeutic target for synucleinopathies. Antibodies that sequester alpha-synuclein aggregates are currently under development, as well as small molecules that bind monomer and prevent primary aggregation (20). We recently identified several rationally designed peptides and small proteins capable of binding to the growing ends of alpha-synuclein fibrils and preventing fibril elongation and seeding (21, 22). In this current study, we present small molecules capable of disaggregating preformed alpha-synuclein fibrils. We demonstrate the efficacy of these molecules with in vitro studies, cell culture models, and in vivo models using both recombinant and patient brain-derived alpha-synuclein fibrils. We also present a structure-based model to describe their possible mechanism of fibril disaggregation.

## Results

The flavonoid epigallocatechin gallate (EGCG) has been previously demonstrated to disaggregate preformed amyloid fibrils (23, 24). In our recent work, we determined the structure of EGCG in complex with tau paired helical filaments (PHFs) extracted from Alzheimer’s disease patient brains (25). From this structure, we identified the pharmacophore of EGCG and proposed EGCG's possible mechanism of disaggregation. Using the EGCG pharmacophore as a docking site, we computationally screened a library of ~60,000 small molecules predicted to have favorable central nervous system penetration based on their biophysical properties (i.e., polar surface area and number of rotatable bonds, etc.). From this screen, we identified compounds able to disassemble tau PHFs, among them, the compound we term CNS-11. The ability of EGCG to disaggregate amyloid fibrils is nonspecific as the compound can act upon multiple different amyloid proteins, including tau, alpha-synuclein, and amyloid-beta (23). Thus, we sought to determine whether CNS-11 specifically disaggregates tau fibrils or whether it affects amyloid fibrils more broadly. We observed that CNS-11 and its chemical analog CNS-11g have a robust effect on alpha-synuclein fibrils. In this current study, we characterize these effects using in vitro, in cellulo, in vivo, and in silico approaches.

### CNS-11 and CNS-11g Disaggregate Recombinant Alpha-Synuclein Fibrils In Vitro.

Following an initial observation that CNS-11 may disaggregate alpha-synuclein fibrils, we tested 10 chemical analogs of CNS-11 to assess whether they have similar properties (*SI Appendix*, Fig. S1A). CNS-11 has a central amide-like backbone within its structure, and the 10 analogs tested (CNS-11a through CNS-11j) also share this core motif. From our preliminary screen using recombinant alpha-synuclein fibrils, we observe that fibrils treated with the analog CNS-11g have a large reduction in insoluble alpha-synuclein as assessed by western blot (*SI Appendix*, Fig. S1B).

**Fig. 1.**
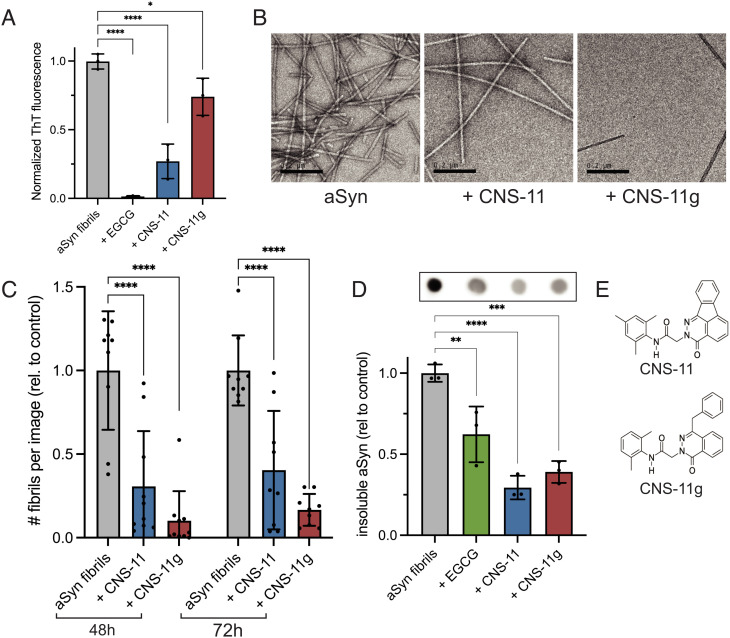
In vitro characterization of alpha-synuclein fibril disaggregation. (*A*) Thioflavin T fluorescence of alpha-synuclein fibrils incubated with equimolar EGCG, CNS-11, or CNS-11g for 48 h. A reduction in ThT signal is observed for compound-treated samples, indicating a reduction in alpha-synuclein fibrils. (*B*) Representative transmission electron micrographs of alpha-synuclein fibrils with CNS-11 and CNS-11g showing a reduction in fibril count after compound treatment. (Scale bar, 200 nm.) (*C*) Quantification of alpha-synuclein fibrils from TEM images after 48- and 72-h compound incubation. N = 10 images from random regions of TEM grids were quantified for each treatment condition. (*D*) Alpha-synuclein fibrils treated with CNS-11 and CNS-11g were pelleted into insoluble fractions and then analyzed by dot blot staining for alpha-synuclein. Blots were quantified, showing a reduction in insoluble alpha-synuclein following compound treatment. (*E*) Chemical structures of CNS-11 and CNS-11g. All error bars represent ± SD (**P < 0.01, ***P < 0.001, ****P < 0.0001) using a one-way ANOVA with pairwise *t* test.

We next aimed to better characterize the ability of CNS-11 and CNS-11g to disaggregate alpha-synuclein fibrils in vitro. Both compounds were incubated with equimolar ratios of recombinant alpha-synuclein fibrils (100 µM:100 µM) for 48 h. After incubation, thioflavin T (ThT), a fluorescent marker of amyloid fibrils, was added to the samples, and ThT fluorescence was measured. EGCG was used as a positive control for comparison. A reduction in ThT signal is observed for all three compounds tested (Fig. 1*A*), indicating a reduction in fibril amount. Next, transmission electron microscopy (EM) was performed for each sample. Representative EM images shown in Fig. 1*B* demonstrate a qualitative reduction in fibril count for samples treated with CNS-11 and CNS-11g. To obtain a quantitative evaluation of fibril reduction, fibrils were incubated for 48 and 72 h with compound, then micrographs from random grid points were taken for each sample, and the numbers of fibrils per image were counted (Fig. 1*C*). We observe a reduction in the number of imaged fibrils for both compounds, particularly for CNS-11g. Dot blot analysis, staining for alpha-synuclein, was performed for fibril samples incubated with compounds for 48 h (Fig. 1*D*). Fibrils treated with EGCG, CNS-11, or CNS-11g were centrifuged to separate insoluble fractions, and a significant reduction in insoluble alpha-synuclein is observed for all compounds. Chemical structures of both CNS-11 (N-mesityl-2-(3-oxoindeno[1,2,3-de]phthalazin-2(3H)-yl)acetamide) and CNS-11g (2-(4-benzyl-1-oxo-2(1H)-phthalazinyl)-N-(2,6-dimethylphenyl)acetamide) are shown in Fig. 1*E*, highlighting their similar chemical structures, low number of hydrogen bond acceptors and donors, and low number of rotatable bonds (*SI Appendix*, Table S1). These physical and chemical properties are more predictive of oral bioavailability and central nervous system penetration compared to EGCG, making them more viable candidates for further drug development.

To assess whether either CNS-11 or CNS-111 g influences primary alpha-synuclein aggregation, we performed a ThT aggregation kinetics assay (*SI Appendix*, Fig. S2). Little difference is seen between alpha-synuclein allowed to aggregate in the presence of either CNS-11 or CNS-11g. Preformed alpha-synuclein fibril seeds were added to the alpha-synuclein sample, which resulted in a faster aggregation time. When the fibril seeds were pretreated with CNS-11 or CNS-11g, the increase in aggregation time was partially reduced, presumably mitigating the effects of the preformed fibrils on aggregation time.

### CNS-11 and CNS-11g Prevent Intracellular Seeding of Alpha-Synuclein Fibrils and Mitigate Alpha-Synuclein Cytotoxicity.

Having established the ability of CNS-11 and CNS-11g to disaggregate alpha-synuclein fibrils in vitro, we next sought to test both compounds in cellular models. It is thought that alpha-synuclein pathology propagates throughout the brain via a prion-like seeding mechanism (19, 26–28). Alpha-synuclein aggregates in one cell can fragment and spread to neighboring cells, where they seed the aggregation of soluble protein. Molecules able to disaggregate preformed fibrils may be able to interrupt this process. To that end, we assessed whether both compounds could mitigate intracellular seeding of alpha-synuclein fibrils using alpha-synuclein biosensor cells—HEK293T cells expressing A53T mutant alpha-synuclein fused with yellow fluorescent protein (YFP) (29). At baseline, the fluorescently labeled alpha-synuclein remains soluble within the cell visible as diffuse fluorescence. However, the liposome-mediated transduction of exogenous alpha-synuclein fibrils into the cells induces aggregation of the endogenous protein, and the aggregates can be visualized and quantified as fluorescent puncta within the cell.

**Fig. 2.**
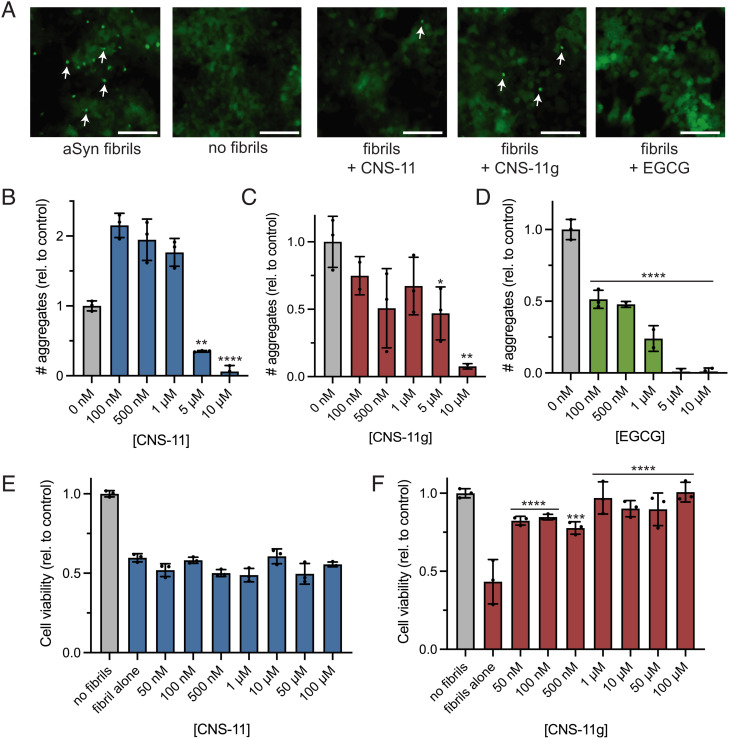
Inhibition of intracellular seeding in alpha-synuclein biosensor cells and mitigation of alpha-synuclein cytotoxicity by CNS-11 and CNS-11g. (*A*) Fluorescent microscopy images of alpha-synuclein biosensor cells with and without 10 µM compound treatment. HEK293T cells expressing YFP-labeled A53T alpha-synuclein, termed biosensor cells, are seeded with exogenous alpha-synuclein fibrils. After seeding, the soluble fluorescent protein is incorporated into intracellular aggregates visible as bright puncta on fluorescence microscopy (“aSyn fibrils,” white arrows). Without addition of fibril seeds, no fluorescent puncta are observed (“no fibrils”). Incubation of CNS-11, CNS-11g, or EGCG with the exogenous fibrils before seeding results in a reduction of visible puncta. (Scale bar, 50 µM.) (*B*–*D*) Quantification of fluorescent puncta from the biosensor cells. Total puncta in each experimental well were quantified from fluorescent microscopy images, which were normalized to total cell confluence. A dose-dependent decrease in puncta with increasing concentrations of compound pretreatment can be seen for CNS-11 (*B*), CNS-11g (*C*), and EGCG (*D*). “No inhibitor” indicates cells seeded with fibrils not treated with any compound. N = 3 experimental replicates were used for each treatment condition. (*E* and *F*) MTT toxicity assay of neuronal cells after treatment with alpha-synuclein fibrils with and without inhibitor. N2a cells were treated with fibrillar alpha-synuclein overnight, resulting in a 40 to 60% reduction in cell viability. CNS-11 was unable to rescue the alpha-synuclein cytotoxicity; however, CNS-11g did show a dose-dependent rescue of cell viability with increasing concentrations of compound. “No fibrils” indicates untreated cells. N = 3 experimental replicates were used for each treatment condition. Error bars represent ± SD.

CNS-11 and CNS-11g at various concentrations were incubated with recombinant alpha-synuclein fibrils for 48 h and then transduced into the biosensor cells. We imaged the cells 48 h after transduction of the samples and quantified the number of fluorescent puncta per sample well. For samples treated with fibrils alone (Fig. 2*A*), numerous bright puncta can be seen throughout the cells (white arrows). Conversely, without any fibrils added, no puncta can be seen. Incubation of compounds with fibrils at a final compound concentration of 100 nM to 10 µM resulted in a dose-dependent reduction in aggregates. We used EGCG as a positive control, which also showed a dose-dependent reduction (Fig. 2 *B*–*D*). For CNS-11, we observed an initial increase in seeding at low compound concentrations and then a sharp reduction in seeding at higher compound concentrations. The mechanism of this is unclear but may be a result of incomplete disaggregation/fragmentation of the fibrils at the low concentration producing a greater amount of seeding, whereas more complete disaggregation is occurring at the higher concentrations.

Alpha-synuclein aggregates, particularly oligomers, are known to be cytotoxic to neurons (4). Thus, we next determined whether disaggregation of alpha-synuclein aggregates by our compounds mitigates their cytotoxicity (Fig. 2 *E* and *F*). Recombinant alpha-synuclein fibrils were again incubated with various concentrations of CNS-11 and CNS-11g for 48 h. The fibril and compound mixtures were then added to cultured Neuro-2a (N2a) neuronal cells to a final alpha-synuclein fibril concentration of 1 µM. A 3-(4,5-dimethylthiazol-2-yl)-2,5-diphenyltetrazolium bromide (MTT) dye reduction cell viability assay of the treated N2a cells reveals that CNS-11g recovers the cytotoxicity of the alpha-synuclein fibrils at substoichiometric concentrations. However, CNS-11 showed no rescue of toxicity at any concentration.

### Molecular Dynamics (MD)Simulations of CNS-11 and CNS-11g Reveal Possible Mechanism of Alpha-Synuclein Fibril Disaggregation.

Having found that CNS-11 and CNS-11g disaggregate recombinant alpha-synuclein, we sought to determine a putative binding site for CNS-11 and CNS-11g to recombinant alpha-synuclein fibrils and to elucidate the compounds’ possible mechanism for fibril disaggregation. To accomplish this, we performed multiple MD simulations of each compound bound to four possible binding sites along the fibril surface, as well as for an unbound control fibril (*SI Appendix*, Fig. S3), using the alpha-synuclein recombinant fibril structure (PDB: 6cu7) (11). MD simulations were carried out for a total of 40 ns. For each simulation, we measured the distance between the top layer of the alpha-synuclein fibril and the layer just beneath it. In most amyloid fibril structures, a distance of ~4.8 Å is seen between fibril layers corresponding to beta-strand separation within the beta-sheet. Thus, if fibril disaggregation occurs, we expect the interlayer spacing to increase beyond 4.8 Å. For each of the four binding sites assessed, we analyzed the interlayer distance between Cα atoms of the residues adjacent to the compound binding site for both CNS-11 and CNS-11g (*SI Appendix*, Fig. S3*B*). For both site 1 and site 2 found at the N terminus of the fibril core, we observe relative stability of the unbound fibril structure at residues 41 to 45, with little fluctuation between interlayer distance. However, with addition of CNS-11 at site 1, the interlayer distances quickly increase (*SI Appendix*, Fig. S3*C*). Similarly, both CNS-11 and CNS-11g greatly disrupt intrastrand spacing when bound to site 2. In contrast, we do not observe large interstrand distance increases in any of the residues adjacent to site 3 or site 4 (*SI Appendix*, Fig. S3*C*) when bound to CNS-11 or CNS-11g. From these findings, we conclude that a possible mechanism of fibril disaggregation by CNS-11 and CNS-11g is through destabilization of the N-terminal residues (Gly41–Lys45) of the fibril core, either bound to site 1 or site 2, and less likely through the C terminus (site 3 and site 4). While this provides an initial putative mechanism by which these compounds are functioning, further structural and biophysical characterization will be needed to fully understand their effects on fibril architecture.

### Compounds Disaggregate MSA Brain-Derived Alpha-Synuclein Fibrils and Prevent Their Seeding.

**Fig. 3.**
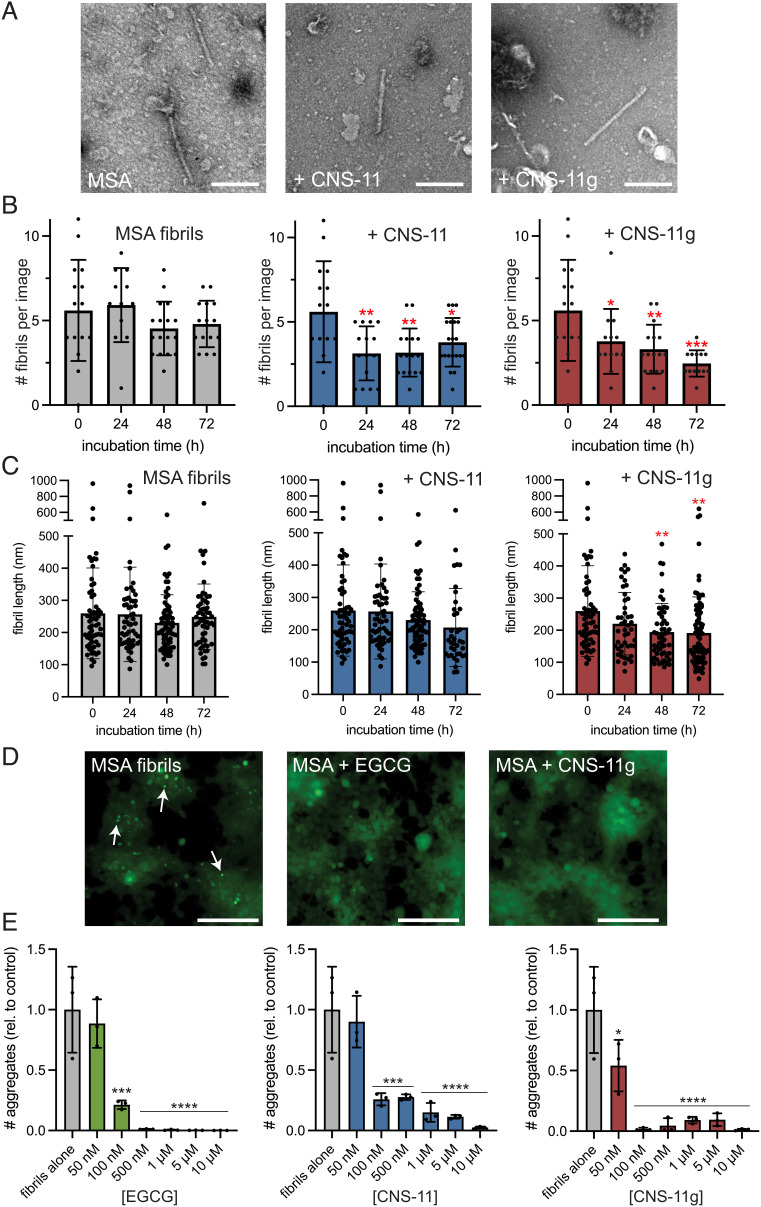
Effects of CNS-11 and CNS-11g on MSA patient brain-derived alpha-synuclein fibrils. (*A*) TEM images of alpha-synuclein fibrils extracted from brains of patients with MSA. (*B*) CNS-11 or CNS-11g was incubated with MSA brain-derived fibrils for up to 72 h. Quantitation of EM images of MSA brain-derived fibrils treated with no compound, CNS-11, or CNS-11g over multiple days. N = 15 images were taken per experimental condition, with each condition performed in triplicate. Average fibril count per image remains relatively stable for the “no inhibitor” control sample, but a reduction in fibrils is seen for both compound-treated samples over the course of 3 d (*P < 0.05, **P < 0.005, ***P < 0.0005). (*C*) Fibril length analysis of MSA brain-extracted fibrils treated with CNS-11 and CNS-11g. Fibrils were incubated with compounds for 3 d, and fibril length was measured every 24 h using transmission EM. A reduction in average fibril length is observed for both CNS-11 and CNS-11g, statistically significant for CNS-11g at 48 and 72-h incubation time. Bars represent mean fibril length; error bars are ±SD. N = 15 TEM images were taken for each condition, with two to eight visible fibrils per image (**P < 0.01). (*D*) Alpha-synuclein biosensor cells seeded with MSA patient-derived alpha-synuclein fibrils. With the addition of the fibril seeds (“+MSA fibrils”), numerous fluorescent puncta are visible (white arrows). Treatment with compound EGCG/CNS-11/CNS-11g greatly reduces the number of puncta visible. (*E*) Quantification of seeded aggregates from MSA fibril–treated biosensor cells. EGCG, CNS-11, and CNS-11g all show a robust effect on reducing intracellular seeding when preincubated with fibril seeds prior to transduction into the cells. N = 3 experimental replicates were analyzed for each treatment condition. Error bars represent ± SD (*P < 0.05, ***P < 0.0005, ****P < 0.0001).

Having found that CNS-11 and CNS-11g disaggregate recombinant alpha-synuclein fibrils, we then investigated the efficacy of each compound on brain-derived alpha-synuclein fibrils. Postmortem alpha-synuclein fibrils were extracted and purified from the brain of a patient with MSA. The presence of fibrils in the extract was confirmed by EM (Fig. 3*A*). The brain-derived fibrils were confirmed to be alpha-synuclein using immunogold labeling (*SI Appendix*, Fig. S4). As with the recombinant fibrils, we next aimed to quantitatively assess brain-derived fibril disaggregation by EM. Compounds were incubated with MSA fibrils for 72 h, and fibrils were quantified by EM daily, with 15 images taken per experimental replicate. We observed a reduction in the average number of imaged fibrils for both CNS-11 and CNS-11g compared to control (Fig. 3*B*). We also observe a reduction in fibril length. For both CNS-11 and CNS-11g, an ~25% reduction in mean fibril length is observed for treated MSA fibrils (Fig. 3*C*). Additional EM images of MSA fibrils used for the analysis are shown in *SI Appendix*, Fig. S5.

Next, we tested the efficacy of the compounds to mitigate the intracellular seeding of MSA fibrils in biosensor cells. MSA brain-derived fibrils robustly seed intracellular alpha-synuclein in HEK293T biosensor cells (Fig. 3*D*) (30). CNS-11, CNS-11g, and EGCG at various concentrations were incubated with MSA brain-derived fibrils for 48 h, sonicated, and then transduced into cells. Cells were imaged after 48 h, and the number of fluorescent puncta per sample well was quantified (Fig. 3*E*). A significant reduction in intracellular seeding is observed for all three compounds at submicromolar concentrations, with both CNS-11 and CNS-11g showing efficacy comparable to the EGCG control. Together, these data demonstrate that these compounds can disaggregate MSA brain-derived fibrils in vitro, and fibrils pretreated with CNS-11 and CNS-11g are no longer competent at seeding the intracellular aggregation of alpha-synuclein.

### CNS-11 and CNS-11g Prevent Aggregation of Alpha-Synuclein in C. elegans.

**Fig. 4.**
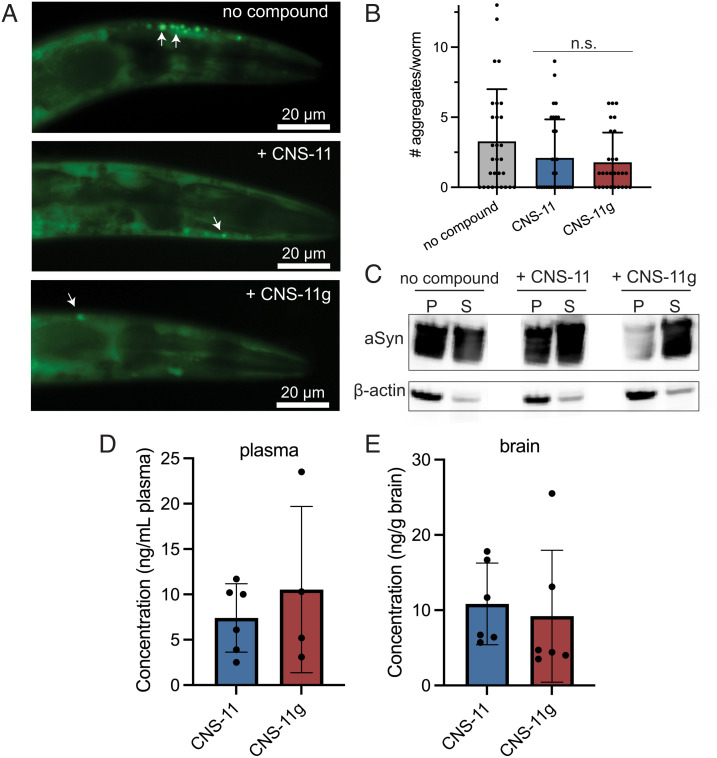
In vivo effects of CNS-11 and CNS-11g in *C. elegans* and mice. (*A*) *C. elegans* overexpressing CFP- and YFP-fused alpha-synuclein were treated with CNS-11 and CNS-11g at the L1 larval stage and then imaged by fluorescent microscopy at day 6 adulthood to assess for alpha-synuclein aggregation. Pseudocolored images show numerous punctate aggregates of fluorescent alpha-synuclein in the head region of vehicle-treated control worms (no inhibitor), and a reduction in aggregates is observed for those treated with either CNS-11 or CNS-11g. (*B*) Quantification of number of aggregates observed in the head region of each treatment group shows a reduction in aggregates for both CNS-11- and CNS-11g–treated worms. (*C*) Untreated and compound-treated *C. elegans* were homogenized, and western blot analysis of insoluble alpha-synuclein [found in sample pellet (P)] and soluble alpha-synuclein [found in sample supernatant (S)] was performed. Without treatment, the majority of alpha-synuclein found in the sample homogenate was found in the insoluble (P) fraction. However, with treatment of either compound, particularly CNS-11g, most of the alpha-synuclein is converted to the soluble (S) fraction. β-actin control is shown below. For each treatment condition, n = 30 worms were analyzed for both the microscopy and western blot analysis. Error bars represent ± SD. (*D* and *E*). Brain penetration of CNS-11 and CNS-11g in mice. Mice were injected intravenously with 1 mg/kg of CNS-11 or CNS-11g (n = 6 per compound) and euthanized 1 h after dosing. Compound levels in plasma (*D*) and brain tissue (*E*) were analyzed using an LC-MS/MS-MRM method.

Given our findings that both CNS-11 and CNS-11g can reduce alpha-synuclein aggregates in vitro and in cellular models, we next studied their efficacy using an in vivo model of alpha-synuclein pathology. The DDP1 *C. elegans* strain overexpresses alpha-synuclein fused with YFP and cyan fluorescent protein (CFP) (31, 32). FRET-positive fluorescent alpha-synuclein aggregates can then be visualized and quantified in the adult worms. This particular model typically shows only modest phenotypic changes or changes in life span. Thus, aggregate count was used as the primary metric to assess compound efficacy. DDP1 worms were synchronized (*Methods*), and then, the L1 larvae were grown on plates treated with CNS-11 or CNS-11g at 100 µM. PBS was used as a vehicle control. At day 6 of adulthood, the worms were visualized, and the number of aggregates was quantified by fluorescent microscopy. As shown in Fig. 4*A*, numerous fluorescent aggregates are visible in the head region of vehicle-treated (“no inhibitor”) worms. Treatment with either compound resulted in a reduction of total aggregates, as quantified in Fig. 4*B*. Treated worms were also homogenized for protein analysis by western blot. Homogenate for each experimental condition was separated into insoluble pellet fractions (P) and soluble supernatant (S) fractions. For untreated worm homogenate, the majority of alpha-synuclein is found in the insoluble pellet. Treatment with CNS-11 and CNS-11g each results in solubilization of the alpha-synuclein, presumably from disrupting the formed aggregates (Fig. 4*C*).

### Detection and Quantification of CNS-11 and CNS-11g in Serum and Brain Tissue in Mice.

Based on their biophysical properties, both CNS-11 and CNS-11g were predicted to have favorable brain permeability. To validate their brain permeability, the compounds were administered by tail vein injection at a dose of 1 mg per kg of body weight to C57BL/6J mice (n = 6 for each compound). One hour after dosing, the mice were killed by cardiac perfusion, and brain and plasma samples were collected. A liquid chromatographic–tandem mass spectrometric multiple reaction monitoring (LC-MS/MS-MRM) assay was used to detect and quantify the drug levels in each tissue sample. The sample extraction protocol for the plasma and brain was optimized with spiking experiments in which the authentic compounds were added to the plasma and brain from drug-naive mice.

One hour following administration, CNS-11 and CNS-11g were measured in the plasma of treated wild-type mice with a range of concentrations of 2.5 to 11.7 ng/mL for CNS-11 and 5.2 to 23.5 ng/mL for CNS-11g (Fig. 4*D*). In addition, both CNS-11 and CNS-11g demonstrated brain permeability. They were measured in brain tissue with a range of concentrations of 5.7 to 17.8 ng/g of brain for CNS-11 and 3.5 to 25.5 ng/g of brain for CNS-11g (Fig. 4*E*). The limit of detection in plasma was determined to be 3.2 ng/mL for CNS-11 and 1.4 ng/mL for CNS-11g. The limit of detection in brain was determined to be 2.9 ng/g for CNS-11 and 2.6 ng/g for CNS-11g. The amount of plasma collected from two mice treated with CNS-11g was an insufficient volume for analysis by LC-MS/MS-MRM.

## Discussion

Current treatment options for synucleinopathies, including PD, MSA, and DLB, are only capable of symptom management; there are no available therapies for any synucleinopathy that modify disease progression. PD is the second most common neurodegenerative disease, and with the aging of our population, the need for therapeutic options is becoming increasingly urgent. Alpha-synuclein was determined to be the primary component of Lewy bodies over 20 y ago (33). Since that discovery, the aggregation of alpha-synuclein has been identified as the key component in the pathology of these diseases (6). Considerable effort has been invested in the screening and identification of compounds able to either inhibit the formation of or disassemble alpha-synuclein aggregates (20). High-throughput small-molecule screens have identified several promising compounds, including SynuClean-D (34), anle138b (35), and BIOD303 (36). Recently, the carotenoid crocin was demonstrated to both inhibit aggregation and disassemble mature alpha-synuclein fibrils (37).

As previously mentioned, CNS-11 was originally identified from a screen of compounds capable of disaggregating tau PHFs, the primary tau aggregate in Alzheimer’s disease (25). This screen was guided by the structure of EGCG bound to disaggregating tau PHFs. EGCG is a known potent disaggregator of amyloid fibrils, including tau (24) and alpha-synuclein (38). Unfortunately, its highly polar chemical composition greatly reduces its bioavailability, diminishing its promise as a potential therapeutic (39). Our aim was to identify compounds capable of disaggregating fibrils like EGCG but with more drug-like biophysical properties. CNS-11 and CNS-11g were identified from a library of compounds with increased probability of blood–brain barrier penetration, a key hurdle in the development of therapeutics for neurodegenerative disease. Both CNS-11 and CNS-11g lack the numerous hydroxyl groups of EGCG, but have low polar surface areas and molecular weights predictive of central nervous system penetration (40), making them much more promising therapeutic leads than EGCG. Both CNS-11 and CNS-11g also satisfy the Lipinski and Veber rules of drug-like compounds, which EGCG violates (*SI Appendix*, Table S1), and are demonstrated to penetrate brain tissue in mice following tail vein injection (*SI Appendix*, Fig. S5).

Here, we have demonstrated that CNS-11 and CNS-11g can disaggregate alpha-synuclein and that treatment of fibrils with either compound also reduces the seeded aggregation of alpha-synuclein. This has important implications as the spread of alpha-synuclein pathology in the brain is thought to occur through a templated seeding mechanism. A possible pitfall of disaggregation as a therapeutic mechanism is that it may fragment fibrils, producing more seeds that can propagate and worsen disease pathology instead of mitigating it. For example, Nachman et al. recently demonstrated that disassembly of tau fibrils by the chaperone protein Hsp70 can generate seeding-competent species (41). Thus, for the investigation of any new potential therapeutic that modifies alpha-synuclein aggregates, it is essential to assess its effects on seeding. In the case of CNS-11, we do observe an increase in seeding of recombinant alpha-synuclein fibrils (Fig. 2) at low compound concentrations, which may represent incomplete disaggregation of the fibrils. This is not seen for CNS-11g, which may indicate that CNS-11g converts the fibrils into a monomeric form or small multimeric form incapable of seeding. We do not observe any enhancement of seeding from MSA brain-extracted fibrils for either compound and instead see a dose-dependent reduction in seeding even at low concentrations. Nonetheless, effects on seeding at a range of doses should be an important consideration during advancement of molecules that target alpha-synuclein, including the compounds presented in this work. We also observe that the toxicity protection by CNS-11g happens at a different concentration range than the reduction in cellular seeding (Fig. 2). This is likely a reflection of different experimental conditions between the two cell-based experiments as different cell lines are used (HEK293 vs Neuro-2a cells).

The cell has existing machinery to combat protein aggregation and disassemble pathologic fibrils once they form. Chaperone proteins Hsc70 with DNAJB1 and Apg2 are able to revert fibrillar alpha-synuclein back to soluble monomer and Hsp70 with DNAJB1 (42). Recent mechanistic studies have revealed that this disassembly of fibrils may occur through the removal of monomer units directly from the fibril ends (43). Our MD experiments of CNS-11 and CNS-11g bound to the recombinant alpha-synuclein fibril core may reveal a similar mechanism of disassembly (Fig. 4). First, our simulations of the compounds docked to four potential binding sites reveal areas near the N terminus of the fibril to be the most destabilized by the compounds. The N terminus of alpha-synuclein has been previously demonstrated as having a strong influence on fibrillization (44). For the simulations that showed fibril disaggregation, we observe that the top layers of the fibrils are the most destabilized even though the simulations are initiated with the compounds bound centrally along the side of the fibril. Thus, CNS-11 and CNS-11g may be acting in a similar way as the chaperone assemblies, peeling off the unstable end layers of the fibrils (*SI Appendix*, Fig. S6). Based on our calculations, the mechanism of binding for either compound to the fibril appears to be driven primarily by interactions with charged side chains, as well as hydrogen bonding with backbone amide groups. However, further structural and biochemical studies will be needed to fully deduce their precise mechanism of action. It should also be noted that several dozen different polymorphic structures of alpha-synuclein have been reported in the literature thus far. Common among many of these structures is a conserved protein fold in the core region of the alpha-synuclein fibril present in both the recombinant and MSA folds. Thus, we sought to use the recombinant fibril structure as a generalized model for our MD simulations as it possesses this conserved core region.

Here, we have demonstrated the ability of two compounds to disassemble alpha-synuclein with both in vitro and in vivo models. We also show the compounds are effective on alpha-synuclein fibrils directly extracted from patient brain tissue and are capable of penetrating living brain tissue in mice. Further validation will be needed to establish their therapeutic efficacy, but these preliminary results demonstrate the potential of these compounds as leads for future drug development toward the treatment of synucleinopathies. Given that the compounds also have effects on tau aggregates and the biophysical properties of a drug-like compound, CNS-11 and its related analogs may also have future promise in treatment of other diseases involving aberrant protein aggregation.

## Methods

### Expression and Purification of Alpha-Synuclein.

The alpha-synuclein construct was transformed into BL21(DE3) Gold *E. Coli*. Protein was expressed following inoculation of a 30 mL starter with colonies after selection with ampicillin. 6L LB medium was inoculated with the starter culture and grown to an OD600 of 0.6 to 0.8 shaking at 220 rpm at 37 °C. Protein expression was induced by the addition of 500 µM IPTG, and then, cells were grown for an additional 3 h. Cells were centrifuged at 4,000 rpm for 5 min, and cell pellet was resuspended in 100 mL of lysis buffer (100 mM Tris-HCl pH 8.0 and 1 mM EDTA), and the pellet was sonicated on ice to lyse cells. Cell lysate was centrifuged for 30 min at 15,000 rpm. Ammonium sulfate of 0.22 g/mL was added to lysate for 30 min and then centrifuged for 30 min at 15,000 rpm. The pellet was resuspended in 80 mL of 20 mM Tris pH 8.0. Solution was dialyzed in 20 mM Tris pH 8.0 overnight. A HiPrep Q HP column (GE Healthcare) was then used to purify the protein. A gradient 0-100% of buffer A (20 mM Tris pH 8.0) to buffer B (20 mM Tris pH 8.0 and 500 mM NaCl) was used over a volume of 100 mL. Fractions were collected and then ran on a size-exclusion G3000 column (Tosoh Bioscience) using a buffer of 100 mM sodium sulfate, 25 mM sodium phosphate, and 1 mM sodium azide at pH 6.5. The protein was then dialyzed in 100 mM sodium sulfate and 25 mM sodium phosphate overnight, with two exchanges of buffer. Protein was concentrated and flash frozen for storage using liquid nitrogen. Concentration of protein was determined by a Pierce BCA assay (Thermo #23225).

### Generation of Recombinant Alpha-Synuclein Fibrils.

First, 50 µM of recombinant alpha-synuclein in 1× PBS was added to several wells of a Nunc black optical bottom plate (Thermo Scientific) to a total volume of 100 μL per well with a single PTFE bead (0.125 inch diameter) per well. Then, the plate was agitated using a Torrey Pines floor shaker on maximum speed for 96 h at 37 °C. The presence of fibrils within the samples was confirmed using transmission EM (see below) prior to use in experimental assays.

### Transmission EM.

For all transmission electron microscopy (TEM), 6 μL of sample was added onto 400 mesh Formvar/carbon film copper mesh grids (EM Sciences) and then incubated for 4 min. Then, 6 μL of 2% uranyl acetate solution was used to stain the grids. After 2 min, excess solution was blotted off, and grids were left to dry for a minimum of 30 min. TEM images were taken on a Tecnai 12 transmission electron microscope. For quantitation of fibrils, n = 20 TEM images were taken per experimental condition from random points throughout the grid. Fibril counts were manually quantified per image in a blinded fashion.

### Immunogold Labeling and EM of MSA Brain-Derived Alpha-Synuclein Fibrils.

Carbon/Formvar 400 mesh copper grids were glow discharged at 15 mA for 30 s. MSA brain fibrillar extract diluted 1:6 in PBS was applied to each of two grids for 3 min. After blotting, grids were blocked with 0.1 % gelatin in PBS for 10 min and incubated with a 1:100 dilution of primary antibody, LB509 (Santa Cruz Biotechnology) in 0.1% gelatin in PBS or just 0.1% gelatin in PBS (control) for 1 h. After five washes in 0.1% gelatin in PBS, the secondary antibody, goat anti-mouse IgG gold (6 nm) (Abcam), was applied to the grids for 30 min. After five washes in water, the grids were stained with 2% uranyl acetate and dried. Grids were imaged at 18,500 to 49,000 times magnification on a FEI Tecnai electron microscope operating at 120 kV.

### Disaggregation Assays.

For all disaggregation assays, 50 mM stocks of CNS-11 and CNS-11g in DMSO were used to make working concentrations of compound in 1× PBS. Compounds were incubated quiescently with fibril samples (both recombinant and brain derived) for 72 h at 37 °C. For ThT measurements, thioflavin T (ThT) was added to samples at 50 µM, and measurements were taken in a Nunc black 96-well optical bottom plate with a FLUOstar OMEGA plate reader (BMG Labtech).

### In Vitro Aggregation Assays.

Thioflavin T (ThT)–based aggregation kinetics assays were performed in Nunc black 96-well optical bottom plates (Thermo Scientific) in a microplate reader taking fluorescence measurements every 10 min (FLUOstar OMEGA, BMG Labtech). All assays were carried out at 37 °C in 1× PBS buffer and 50 µM ThT at a final well volume of 100 μL; 50 µM full-length αSyn was used. PTFE beads (0.125 inch diameter) were used to agitate the sample, and plates were shaken at 700 rpm with double orbital rotation. For compound-treated samples, 50 µM CNS-11 and CNS-11g dissolved in PBS were used. For fibril-seeded samples, 2.5 µL of fibrils was added to each well (at 50 µM original alpha-synuclein concentration).

### Western and Dot Blot Analysis.

Samples were loaded onto a NuPAGE 12% Bis-Tris precast protein gel and ran for 35 min at 200 V. To transfer protein from the gel to a nitrocellulose membrane, an iBLOT2 dry blotting system was used. The membrane was blocked in TBST with 5% milk for 1 h and then washed three times with TBST. The membrane was incubated with the primary antibody (anti–alpha-synuclein MJFR1 (Abcam, catalog #ab138501); 1:5,000 dilution in 5% milk/TBST solution) for 1 h, washed three times with TBST, incubated with the horseradish peroxidase–conjugated secondary antibody (goat anti-rabbit IgG H + L (Invitrogen; catalog #A27036, lot #2116291); 1:4,000 dilution in 5% milk/TBST), and washed three times in TBST. Signal was detected with a Pierce ECL Plus Western Blotting Substrate (catalog # 32132), and blot was imaged with a Pharos FX Plus Molecular Imager.

For *C. elegans* experiments, the same protocol was used, except following the treatment period worms were harvested, flash frozen, and then homogenized for protein extraction. The membrane was washed with TBST overnight before repeating the western blot analysis for beta-actin (primary antibody anti-beta-actin (C4) (Santa Cruz Biotechnology, catalog #sc-47778, lot #J1119); 1:500 dilution in 5% milk/TBST) and horseradish peroxidase–conjugated secondary antibody (goat polyclonal anti-mouse IgG (Abcam; catalog #ab205719, lot #GR3271082-2); 1:5,000 dilution in 5% milk/TBST).

For dot blots alpha-synuclein fibrils were incubated with equimolar ratios of compounds (100 µM:100 µM) for 48 h at 37 °C in PBS for various lengths of time. Samples were spotted onto the nitrocellulose membrane, and 10 µL was spotted for each condition, spotting 2 µL at a time, and allowed to dry in between. The membrane was blocked in TBST with 5% milk for 1 h and then washed three times with TBST. The membrane was then incubated with primary antibody (anti–alpha-synuclein MJFR1 (Abcam, catalog #ab138501); 1:4,000 dilution in 2% milk/TBST solution for 1 h), washed three times with TBST, incubated with the horseradish peroxidase–conjugated secondary antibody (goat anti-rabbit IgG H + L (Invitrogen; catalog #A27036, lot #2116291); 1:5,000 dilution in 2% milk/TBST), and washed three times in TBST. Signal was detected with a Pierce ECL Plus Western Blotting Substrate (catalog # 32132), and the blot was imaged with a Pharos FX Plus Molecular Imager. Densitometry analysis was performed using ImageJ.

### 3-(4,5-dimethylthiazol-2-yl)-2,5-diphenyltetrazolium Bromide (MTT) Dye Reduction Cell Viability Assay.

Neuro-2a cells (ATCC catalog # CCL-131) were cultured in MEM media (Life Technologies catalog # 11095-080) with 10% FBS (Life Technologies catalog # 10437010) and 1% penicillin–streptomycin (Life Technologies catalog # 15140122) in a 5% CO_2_ incubator at 37 °C. N2a cells were plated onto clear 96-well plates (Costar catalog # 3596) at 5,000 cells/well in 90 μL culture media for 24 h. Recombinant alpha-synuclein fibril samples were coincubated with and without CNS-11 or CNS-11g in 10 μL volume overnight at 37 °C and then added to the N2a cells (final fibril concentration of 1 µM). All experiments were performed in triplicate. After incubation for 24 h, 20 μL thiazolyl blue tetrazolium bromide MTT dye (Sigma; 5 mg/mL stock in DPBS) was added to each well and then incubated for 3.5 h at 37 °C. Removal from the incubator and replacement of well media with 100 µL of 100% DMSO halted the assay. Absorbance was measured at 570 nm using a SpectraMax M5 reader. A background reading at 700 nm was subtracted from the 570 nm reading. Well readings were normalized to vehicle-alone–treated cells (designated as 100% viable) and cells treated with 100% DMSO (designated as 0% viable).

### Biosensor Cell Seeding Assays.

HEK293T biosensor cells stably expressing YFP-fused A53T mutant alpha-synuclein, developed and provided by the laboratory of Marc Diamond at the UTSW, were used. Cells were grown in DMEM (Life Technologies, catalog 11965092) with FBS (10% vol/vol; Life Technologies, catalog A3160401), penicillin/streptomycin (1%; Life Technologies, catalog 15140122), and GlutaMAX (1%; Life Technologies, catalog 35050061) at 37 °C and 5% CO_2_ in a humidified incubator. Compounds were incubated with recombinant or patient-derived alpha-synuclein fibrils for 48 h in Opti-MEM media and then applied to ~70% confluent biosensor cells. Prior to adding to cells, the coincubated compound/fibril solution was sonicated for 5 min in a Cup Horn water bath and then mixed for 20 min with Lipofectamine 2000 in Opti-MEM (1:20 dilution) for 20 min. 10 μL of the inhibitor/fibril + Lipofectamine mixture was added to 90 μL of cells plated in black 96-well tissue culture plates in triplicate for each concentration of compound tested. The number of seeded aggregates was quantified using a Celigo Image Cytometer (Nexcelom) in the YFP channel. Images were processed in ImageJ; background fluorescence from unseeded cells was subtracted, and the number of particles per image was counted using the particle analyzer function. The quantity of aggregates in each well was normalized to cell confluence. SD between triplicates and a nonlinear regression curve was used to calculate IC_50_ values for dose–response curves. High-quality fluorescent images were obtained using a ZEISS Axio Observer D1 fluorescence microscope in the YFP channel.

### Extraction of Alpha-Synuclein Fibrils from Patient Brain Tissue.

Extraction of sarkosyl-insoluble alpha-synuclein fibrils from neuropathologically confirmed brain samples of patients diagnosed with MSA was performed using the method previously described by Schweighauser et al. without any modifications (17). The presence of fibrils in each extract was confirmed by TEM prior to use in experiments. The presence of alpha-synuclein was confirmed by immunoblotting using anti–alpha-synuclein MJFR1 antibody (Abcam, catalog #ab138501).

### C. elegans Experiments.

The DDP1 (uonEx1 [unc-54::αSyn::CFP + unc-54::αSyn::YFP(Venus)]) strain was acquired from the Caenorhabditis Genetics Center (CGC) and used for experiments. *C. elegans* were grown and maintained using standard conditions. Worms were synchronized using hypochlorite bleaching, hatched overnight in M9 media (5 g/L NaCl, 6 g/L Na_2_HPO_4_, 3 g/L KH_2_PO_4_, and 1 µM MgSO_4_) at 17 °C, and then cultured on plates with nematode growth medium (NGM; 17 g/L agar, 2.5 g/L peptone, 3 g/L NaCl, 1 mM CaCl_2_, 1 mM MgSO_4_, 25 mM KH_2_PO_4_ pH 6, and 5 µg/mL cholesterol) seeded with OP50 *E. coli*. Strains were maintained at 17 °C. For inhibitor treatment conditions, CNS-11 and CNS-11g were diluted in 1× PBS to a final compound concentration of 100 µM, and the solution was added to plates. Treatment plates were then seeded with heat-treated OP50 (30 min at 65 °C), according to the “NGM dead” method as previously published (45). Synchronized L1 worms were then added to the treatment plates and grown for 7 d. PBS was used as a control condition. On the third day of growth, 75 µM FUDR was added. For imaging, worms were mounted onto 5% agar pads on glass slides, immobilized with 1% NaN_3_ solution, and imaged by fluorescent microscopy (GFP channel) using a ZEISS Axio Observer D1 fluorescence microscope. Alpha-synuclein aggregates in the head region were quantified.

### Animal Experiments.

All animal experiments were approved by the UCLA Animal Research Committee and performed under oversight of the Division of Laboratory Animal Medicine (DLAM). C57BL/6J mice (Jackson Laboratories: JAX:000664) were housed on a 12-h light–dark schedule.

### Sample Preparation for MRM Analysis.

Mice were injected intravenously with CNS-11 (n = 6) or CNS-11g (n = 6) at a concentration of 1 mg/kg and euthanized by perfusion 1 h postinjection. Blood was collected by cardiac puncture, and plasma was recovered as the supernatant after centrifugation (3,000 × g, 10 m) and stored frozen. Brains were collected by standard dissection and immediately frozen.

To measure the average drug concentrations across the entire brains, each left hemisphere (average wet weight 250 mg) was processed. The samples were spiked with the internal standard (IS, CNS-11F, 15 pmol in 15 µL of acetonitrile) and homogenized with a probe sonicator (Kontes microsonic cell disrupter, 30 s) after the addition of acetonitrile/water (4:1, v/v, 2,500 µL). The homogenates were divided evenly into three polypropylene microcentrifuge tubes (mcts) for triplicate measurements of each sample. Following centrifugation (16,100 g, 5 min), the supernatants were transferred to clean mcts and dried in a vacuum centrifuge. The pellets were treated with acetonitrile (25 µL) with vigorous mixing, followed by water (25 µL) with more vigorous mixing, and followed by more water (50 µL) for a total volume of 100 µL. The samples were mixed again, centrifuged (16,100 g, 5 min), and the supernatants were transferred to HPLC injector vials. With each batch of tissue samples, a series of standards were prepared in which CNS-11 and CNS-11g (0, 1, 2.5, 5, and 10 pmol, each in duplicate) were added to drug-naive perfused murine brain tissue (100 mg per tube) along with the same amount of IS (5 pmoles). These samples were processed as described above.

Plasma samples (50 µL, in duplicate for each mouse) were spiked with the internal standard (IS, CNS-11F, 5 pmol in 5 µL of acetonitrile) and vigorously mixed after the addition of acetonitrile (500 µL). Following centrifugation (16,100 g, 5 min), the supernatants were transferred to clean mcts and dried in a vacuum centrifuge. The pellets were treated with acetonitrile (25 µL) with vigorous mixing, followed by water (25 µL) with more vigorous mixing, and followed by more water (50 µL) for a total volume of 100 μL. The samples were mixed again and centrifuged (16,100 g, 5 min), and the supernatants were transferred to HPLC injector vials. With each batch of plasma samples, a series of standards were prepared in which CNS-11 and CNS-11g (0, 1, 2.5, 5, and 10 pmol, each in duplicate) were added to drug-naive murine plasma (50 µL per tube) along with the same amount of IS (5 pmoles). These samples were processed as described above.

### Combined Liquid Chromatography–Tandem Mass Spectrometry with Multiple Reaction Monitoring (MRM).

Aliquots of each sample (2 µL) were injected onto a reverse-phase HPLC column (Phenomenex Kinetex C18, 2.6 µm, 100 × 2.1mm) equilibrated in solvent A (water/formic acid, 100/0.1, v/v) and eluted (100 µL/min) with a linearly increasing concentration of solvent B (acetonitrile/formic acid, 100/0.1, v/v; min/%B: 0/5, 5/5, 30/100, 32/5, and 40/5). The effluent from the column was directed to an electrospray ion source (Agilent Jet Stream) connected to a triple quadrupole mass spectrometer (Agilent 6460) operating in the positive ion tandem mass spectrometric multiple reaction monitoring (LC-MS/MS-MRM) mode in which the intensity of the transition of preselected parent ions to preselected fragment ions was recorded after signal optimization (collision energy, fragmentor voltage, and collision cell accelerator voltage) with instrument manufacturer-supplied software (Mass Hunter). Two transitions were monitored for each drug: m/z 398.1 → 249.2 (for quantitation) and 277.2 (for confirmation) for CNS-11G (retention time 23.3 min); m/z 396.2 → 233.1 (for quantitation) and 261.0 (for confirmation) for CNS-11 (retention time 24.3 min), and one transition was monitored for the IS; m/z 412.3 → 249.2 (retention time 24.3 min). Peak areas were integrated and recorded, and a curve was constructed from the data obtained from the standards in which the ratio of drug peak area/IS peak area was plotted against the amount of drug in each sample. The amount of each drug in the samples was then derived by interpolation from the curve.

### Computational Models and MD.

Three-dimensional structures of CNS-11 and CNS-11g were generated using Open Babel. Both compounds were docked to four unique locations along the alpha-synuclein fibril structure (PDB code: 6cu7) using AutoDock vina. Top-scoring bound poses were used as starting trajectories for subsequent MD experiments. MD was performed using GROMACS version 2020 using a CHARMM36 force field. Ligand topologies for CNS-11 and CNS-11g were generated using the CGenFF server. Hydrogen atoms were added to all structures using Avogadro. The fibril alone and fibril/compound complex structures were solvated using a 1-nm dodecahedron water box. Chloride ions were added to generate a charge-neutral system. The system was first energy minimized; then, temperature and pressure equilibrated under and NVT ensemble for 100 ps and then NPT for an additional 100 ps. Production MD runs were performed for 40 ns for each system. Analysis of molecular distances was performed in GROMACS and manually visualized with PyMOL. Images of molecular models were generated with UCSF Chimera.

## Supplementary Material

Appendix 01 (PDF)

## Data Availability

All study data are included in the article and/or *SI Appendix*.
