# Supplementary material for: Small molecules disaggregate alpha-synuclein and prevent seeding from patient brain-derived fibrils
Source: Proc Natl Acad Sci U S A. 2023 Feb 9;120(7):e2217835120. doi: 10.1073/pnas.2217835120 (PMC9963379; doi:10.1073/pnas.2217835120)
Supplement: Supplementary file 1 — Appendix 01 (PDF) [file pnas.2217835120.sapp.pdf]

Compound	Chemical name	H-bond donors	H-bond acceptors	Molecular weight	XLogP3	Rotatable bonds	Polar surface area (Å^2)
**EGCG**	Epigallocatechin gallate	8	11	458.4	1.2	4	197
**CNS-11**	N-mesityl-2-(3-oxoindeno[1,2,3-de]phthalazin-2(3H)-yl)acetamide	1	3	395	4.6	3	62
**CNS-11g**	2-(4-Benzyl-1-oxo-2(1H)-phthalazinyl)-N-(2,6-dimethylphenyl)acetamide	1	3	397.5	4.4	5	62

Supplemental Table 1: Physical and chemical properties of alpha-synuclein disaggregator compounds. EGCG, CNS-11, and CNS-11g are listed. Compounds were assessed based on Lipinski's rule for druglikeness (H-bond donors <5, H-bond acceptors <10, MW < 500, lipid partition coefficient (log P) <5) and Verber's rule (rotatable bonds <10, polar surface area <140 Å^2) both of which are predictive of oral bioavailability. Properties that satisfy the rules are colored in green, and those that violate the criteria are colored red. Note that both CNS-11 and CNS-11g satisfy all terms in the rules, while EGCG violates several.

Recovery of CNS-11 and CNS-11G from mouse brain				
**Sample**	**CNS-11G**	**CNS-11G**	**CNS-11**	**CNS-11**
	**398.1-->249.2**	**398.1-->277.2**	**396.2-->233.1**	**396.2-->261.0**
**Id**	**area**	**area**	**area**	**area**
**Br. 1A**	103	96	444	420
**Br. 1B**	141	118	207	244
**Br. 1C**	214	226	398	405
**Br. 2A**	81	111	357	371
**Br. 2B**	136	123	372	436
**Br. 2C**	94	92	366	428
**Br. 3A**	118	136	334	344
**Br. 3B**	67	75	171	163
**Br. 3C**	118	113	170	208
**Br. 4A**	93	87	1829	1794
**Br. 4B**	100	111	1394	1345
**Br. 4C**	93	63	1095	1053
**Br. 5A**	39	21	1167	723
**Br. 5B**	18	15	693	603
**Br. 5C**	26	8	569	611
**Br. 6A**	50	32	283	411
**Br. 6B**	14	19	418	345
**Br. 6C**	17	21	417	528
**Br. 7A**	1669	1812	3	2
**Br. 7B**	1612	1624	7	2
**Br. 7C**	1063	1026	2	2
**Br. 8A**	1702	1743	4	4
**Br. 8B**	1513	1534	3	4
**Br. 8C**	882	912	4	1
**Br. 9A**	1336	1387	2	3
**Br. 9B**	1288	1327	1	2
**Br. 9C**	1183	1106	3	3
**Br. 10A**	9811	9439	1	3
**Br. 10B**	8293	7995	1	2
**Br. 10C**	8970	8965	6	2
**Br. 11A**	19334	18853	7	1
**Br. 11B**	23298	22164	6	4
**Br. 11C**	21003	19632	5	8
**Br. 12A**	2174	1899	5	5
**Br. 12B**	1984	2114	2	4
**Br. 12C**	3369	2984	2	2
**Std. 1 (0 pmoles)**	54	46	7	5
**Std. 2 (0 pmoles)**	40	37	8	7
**Std. 3 (1 pmoles)**	3663	3472	362	410
**Std. 4 (1 pmoles)**	4112	3936	328	316
**Std. 5 (2.5 pmoles)**	8515	8075	997	976
**Std. 6 (2.5 pmoles)**	9382	9094	1181	1101
**Std. 7 (5 pmoles)**	15557	14289	2126	1908
**Std. 8 (5 pmoles)**	18851	17469	2728	2517
**Std. 9 (10 pmoles)**	33825	31045	5016	4919
**Std. 10 (10 pmoles)**	35666	33434	5570	5297

Supplemental Table 2: Raw LC-MS/MS-MRM data mouse experiments, brain tissue.

Recovery of CNS-11 and CNS-11G from mouse plasma						
**Sample**		**CNS-11G**	**CNS-11G**		**CNS-11**	**CNS-11**
		**398.1-->249.2**	**398.1-->277.2**		**396.2-->233.1**	**396.2-->261.0**
**Id**	**rt**	**area**	**area**	**rt**	**area**	**area**
**Pl. 1A**	25.10	270	307	26.10	567	568
**Pl. 1B**	25.10	158	175	26.10	556	545
**Pl. 2A**	25.10	200	214	26.10	611	690
**Pl. 2B**	25.10	528	486	26.10	486	468
**Pl. 3A**	25.10	185	208	26.10	351	383
**Pl. 3B**	25.10	220	232	26.10	306	305
**Pl. 4A**	23.30	17	23	24.30	968	949
**Pl. 4B**	23.30	45	39	24.30	916	885
**Pl. 5A**	23.30	15	24	24.30	134	141
**Pl. 5B**	23.30	13	16	24.30	162	147
**Pl. 6A**	23.30	28	7	24.20	119	94
**Pl. 6B**	23.30	14	17	24.20	115	110
**Pl. 7A**	25.00	2001	1953	27.70	2	1
**Pl. 7B**	25.10	1450	1415	27.70	2	1
**Pl. 10A**	25.00	270	307	26.10	567	568
**Pl. 10B**	25.00	158	175	26.10	556	545
**Pl. 11A**	25.10	200	214	26.10	611	690
**Pl. 11B**	25.00	528	486	26.00	486	468
**Pl. 12A**	23.30	1614	1428	24.30	3	3
**Pl. 12B**	23.30	1341	1189	24.30	1	2
**Std. 1 (0 pmoles)**	23.30	31	23	24.30	20	6
**Std. 2 (0 pmoles)**	23.30	30	24	24.30	8	12
**Std. 3 (1 pmoles)**	23.30	4693	4185	24.30	612	609
**Std. 4 (1 pmoles)**	23.30	5863	5395	24.30	659	569
**Std. 5 (2.5 pmoles)**	23.30	10384	9797	24.30	1332	1176
**Std. 6 (2.5 pmoles)**	23.30	12535	11494	24.30	1326	1269
**Std. 7 (5 pmoles)**	23.30	19349	17894	24.30	1914	1946
**Std. 8 (5 pmoles)**	23.30	19516	18218	24.30	2109	2049
**Std. 9 (10 pmoles)**	23.30	34068	31204	24.30	4776	4567
**Std. 10 (10 pmoles)**	23.30	35587	33056	24.30	4851	4665

Supplemental Table 3: Raw LC-MS/MS-MRM data mouse experiments, plasma.

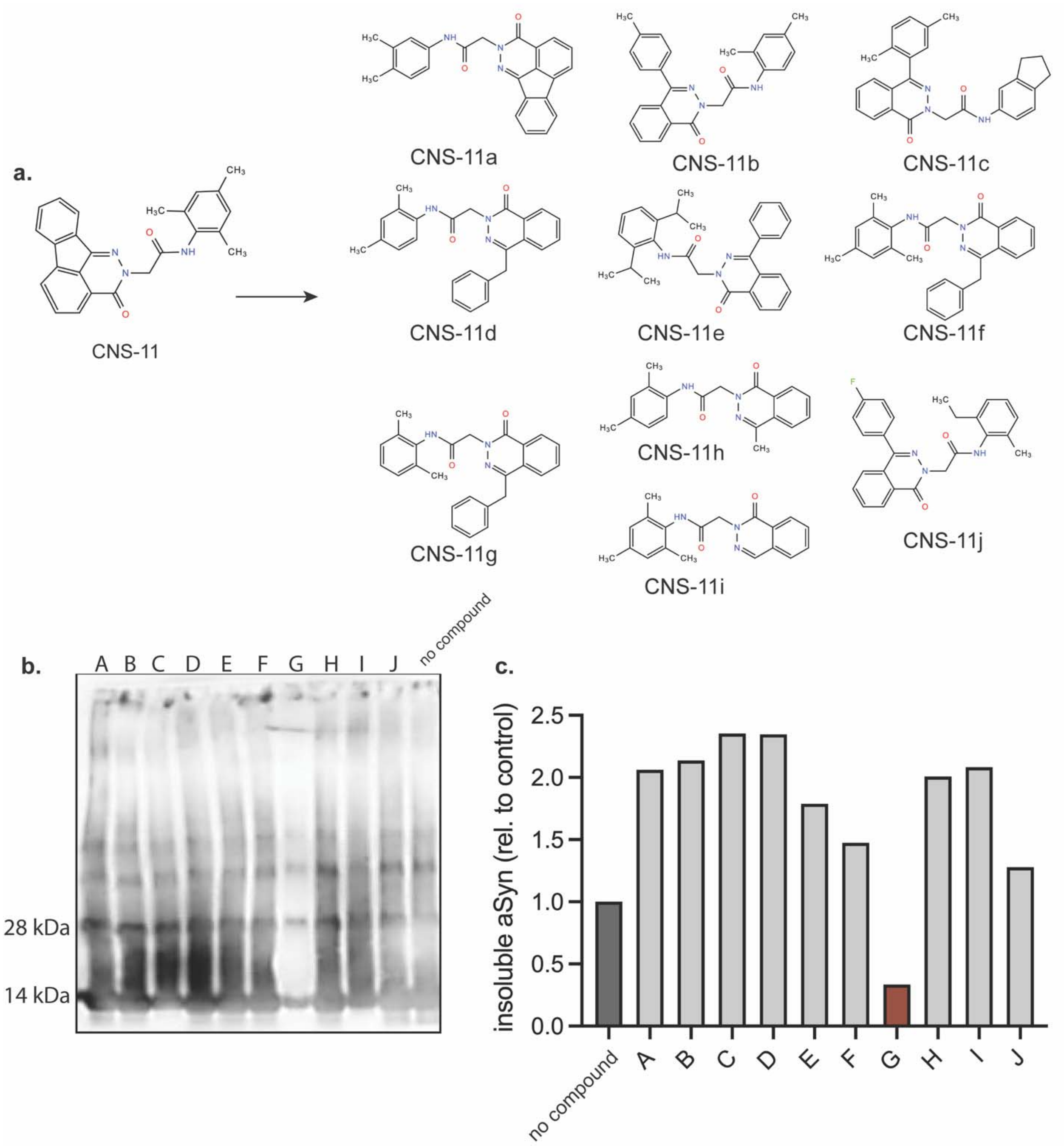

Supplemental Figure 1: Chemical analogs of CNS-11. a. Following the initial observation that CNS-11 was capable of disaggregating alpha-synuclein fibrils, 10 additional chemical analogs were screened, termed CNS-11a through CNS-11j. Notably all compounds maintain the central amide-type backbone found in the original CNS-11 chemical structure. To initially screen the ability of the analog compounds to disaggregate alpha-synuclein, compounds were incubated with recombinant alpha-synuclein fibrils for 72 hours. **b.** Western blot of the insoluble fraction of compound treated fibrils, staining for alpha-synuclein, is shown. Bands for monomeric (14 kDa), dimeric (28 kDa), and larger multimers of alpha-synuclein are visible. **c.** Quantification

of Western blot bands shows a large reduction in insoluble aSyn for CNS-11g treated fibrils (red bar), prompting further experimental investigation.

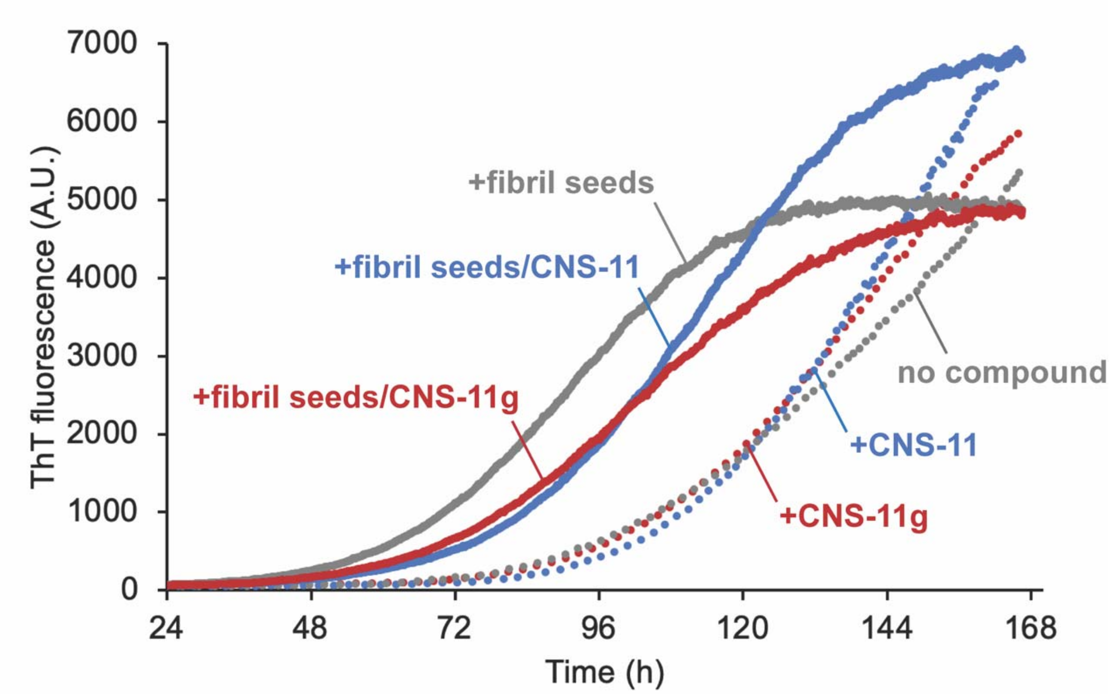

Supplemental Figure 2: Aggregation kinetics of seeded and non-seeded alpha-synuclein treated with compounds CNS-11 and CNS-11g. Monomeric alpha-synuclein (50 µM) was allowed to aggregate with ThT, which fluoresces in the presence of amyloid fibrils. Alpha-synuclein was aggregated without compound (“no compound”; dotted grey line), or in the presence 50 µM CNS-11 (dotted blue line) or CNS-11g (dotted red line). Little difference is seen in the aggregation time for each condition. Pre-formed fibril seeds (2.5 µl at 50 µM aSyn) were added to alpha-synuclein monomer (“+fibril seeds”; solid grey line), resulting in a faster aggregation time, as seen by a large decrease in the aggregation lag time. Fibril seeded samples were also pre-treated with CNS-11 (solid blue line) and CNS-11g (solid red line) for 48 hours then added to the alpha-synuclein monomer, which led to an increase in aggregation time, negating some of the effects of the preformed fibril seeds. N=3 experimental replicates were used per condition.

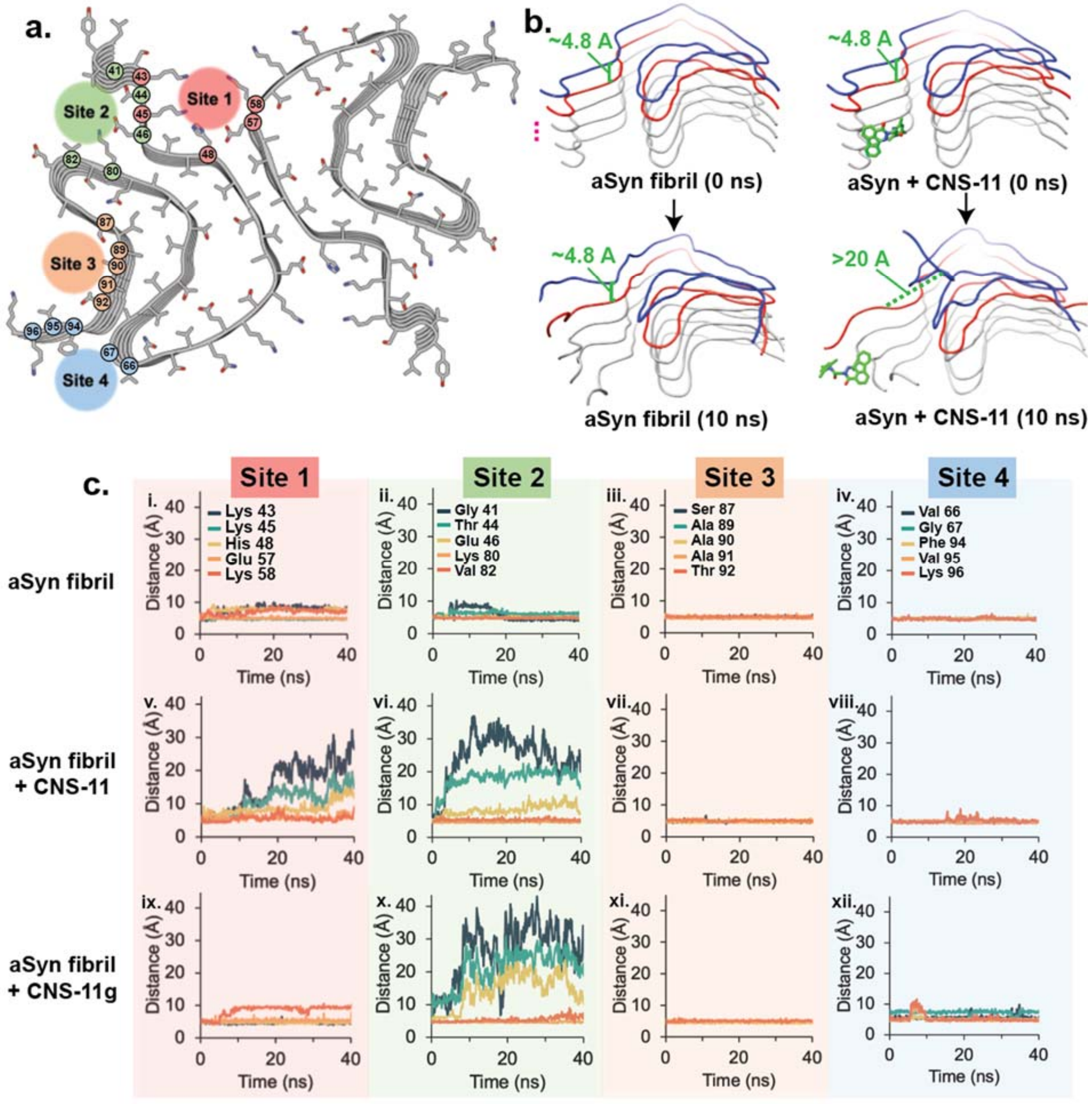

Supplemental Figure 3: Molecular dynamics simulation of CNS-11 and CNS-11g in complex with alpha-synuclein fibril. a. Atomic structure of recombinant alpha-synuclein fibril (PDB code 6cu7). Both CNS-11 and CNS-11g were docked into four potential binding sites along the fibril surface (Site 1 – Site 4) using AutoDock Vina. Key residues at each binding site are labeled (color/numbered circles indicate residue site and number). **b-c.** Molecular dynamics simulations were performed for each compound docked to each of the four unique binding sites of the alpha-synuclein fibril to model fibril disaggregation and intra-strand spacing of the fibril layers was measured at residues within radius of the selected binding site. (**b**) At the beginning of the simulation, spacing between the top two layers of the fibril structure is ~4.8 Å (red and blue strands), the standard inter-strand distance found in amyloid fibrils (top images, “0 ns”). After 10 ns of simulation, the distance between the top two fibril layers is assessed. For the fibril with

no compound bound, the 4.8 Å spacing is generally maintained across all simulations. The addition of compound CNS-11 to Site 2 along the fibril results in a separation between strand layers. (**c**) Measurements of intra-strand spacing for MD simulations of the unbound fibril, or fibril in complex with CNS-11 or CNS-11g performed at the four different compound binding locations. When bound to Site 1, CNS-11 noticeably disrupts the fibril structure, causing an increase in intra-strand spacing. At Site 2, both CNS-11 and CNS-11g lead to fibril disruption, as evidenced by large variations in strand separation over time. When bound to Site 3 or Site 4, very little perturbation is observed for either compound This potentially indicates that the two compounds may be exerting their disaggregating effects at the N-terminus of the fibril core, near Sites 1 and 2, and less likely at the C-terminus near Sites 3 and 4. All MD simulations were performed for a total of 40 ns.

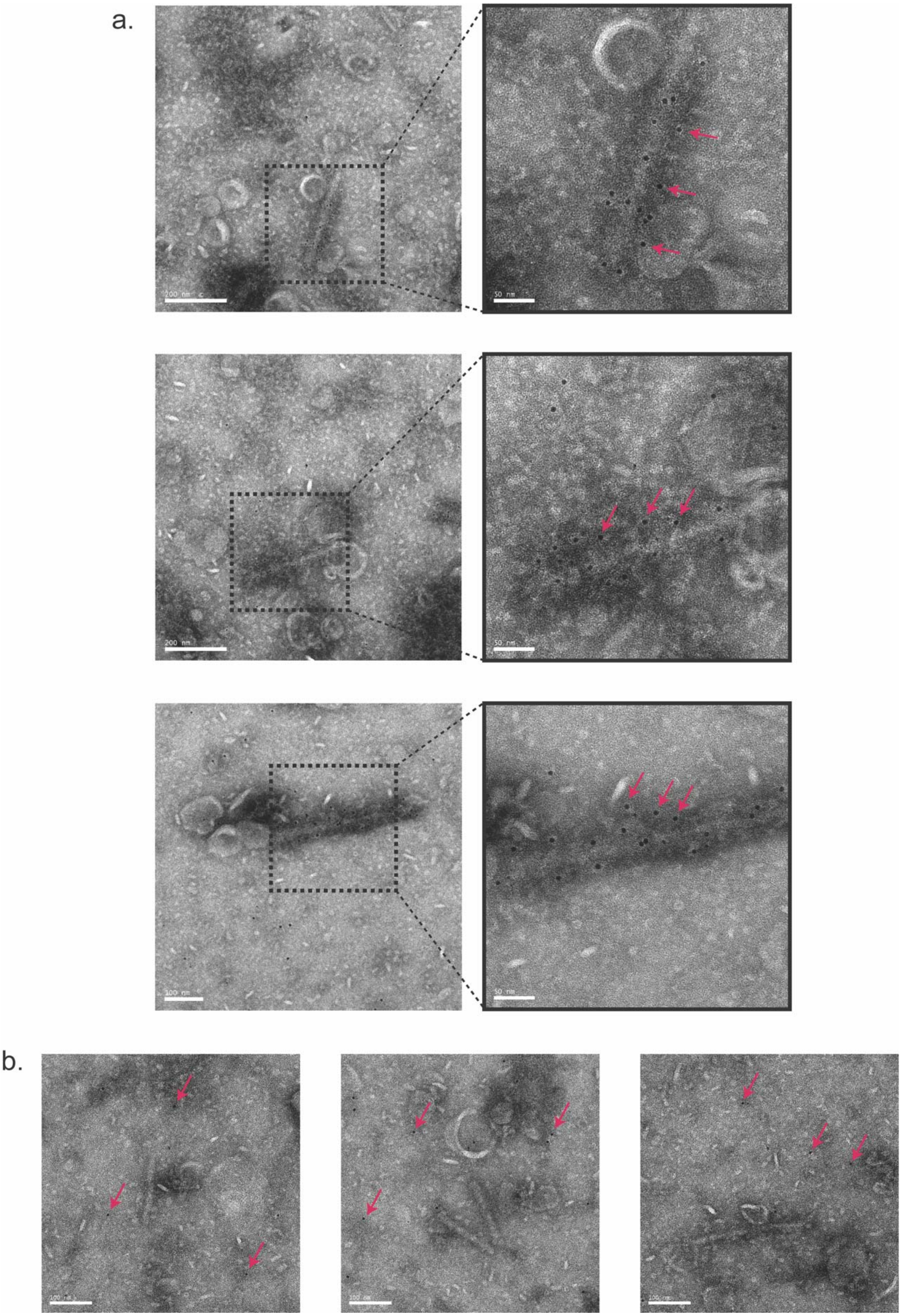

Supplemental Figure 4: Immunogold labeling of MSA patient brain-derived fibrils used for disaggregation studies. a. Alpha-synuclein fibrils extracted from MSA patient

brains labeled with primary alpha-synuclein antibody, LB509, and secondary antibody, Goat pAb to Mouse IgG Conjugated to 6 nm Gold Particles. Binding of numerous immunogold particles (indicated by red arrows) is specific to the extracted fibrils, with little binding observed on other cellular components in the field. **b**. As a negative control, samples were treated without the primary antibody (LB509) and treated only with the secondary immunogold-labeled antibody. Without the primary (alpha-synuclein specific) antibody, immunogold binding is not localized to the fibrils.

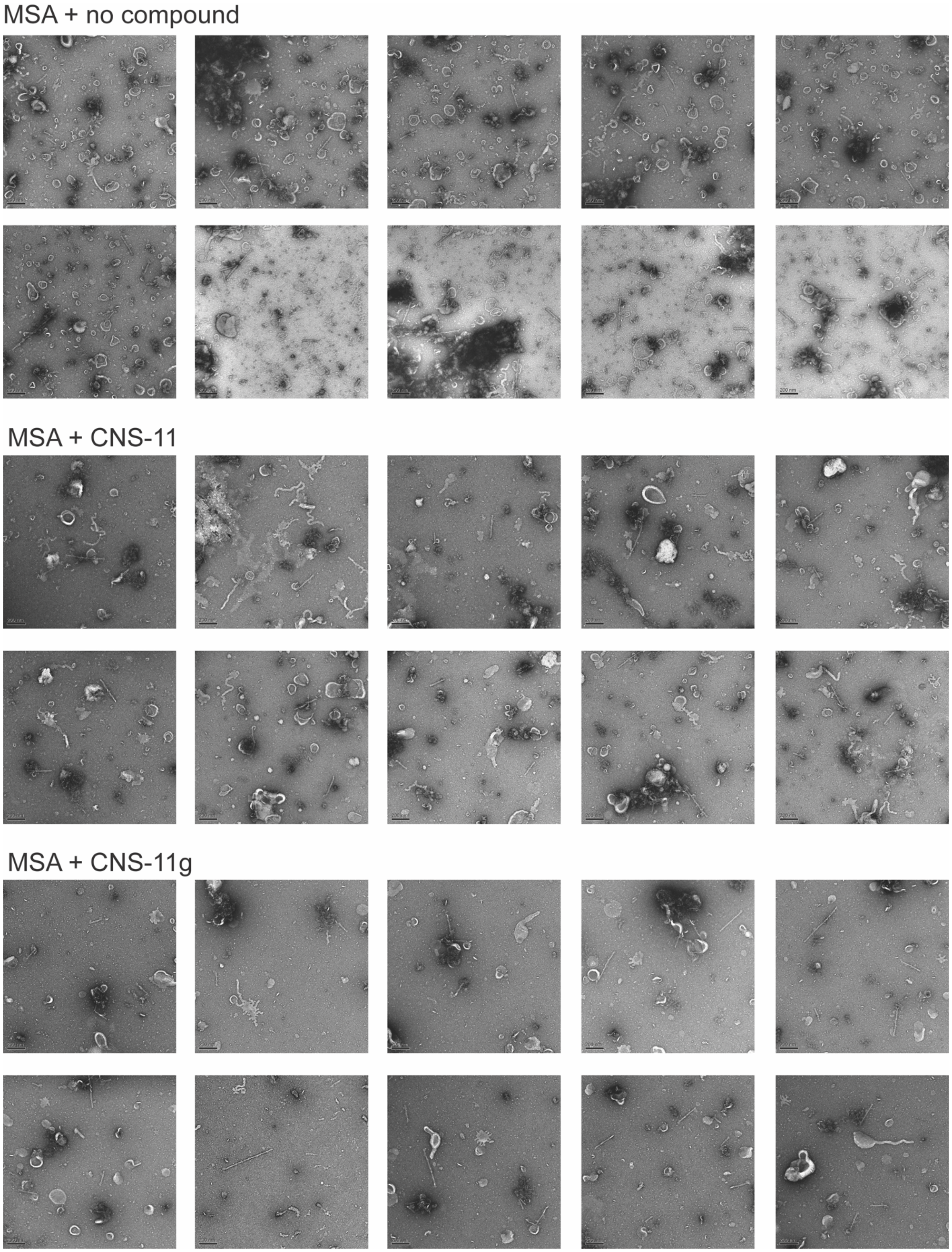

Supplemental Figure 5: Examples TEM used for quantification and length analysis of MSA fibrils. The above images are at 72 hours incubation time of MSA fibrils without compound, with CNS-11 or CNS-11g.

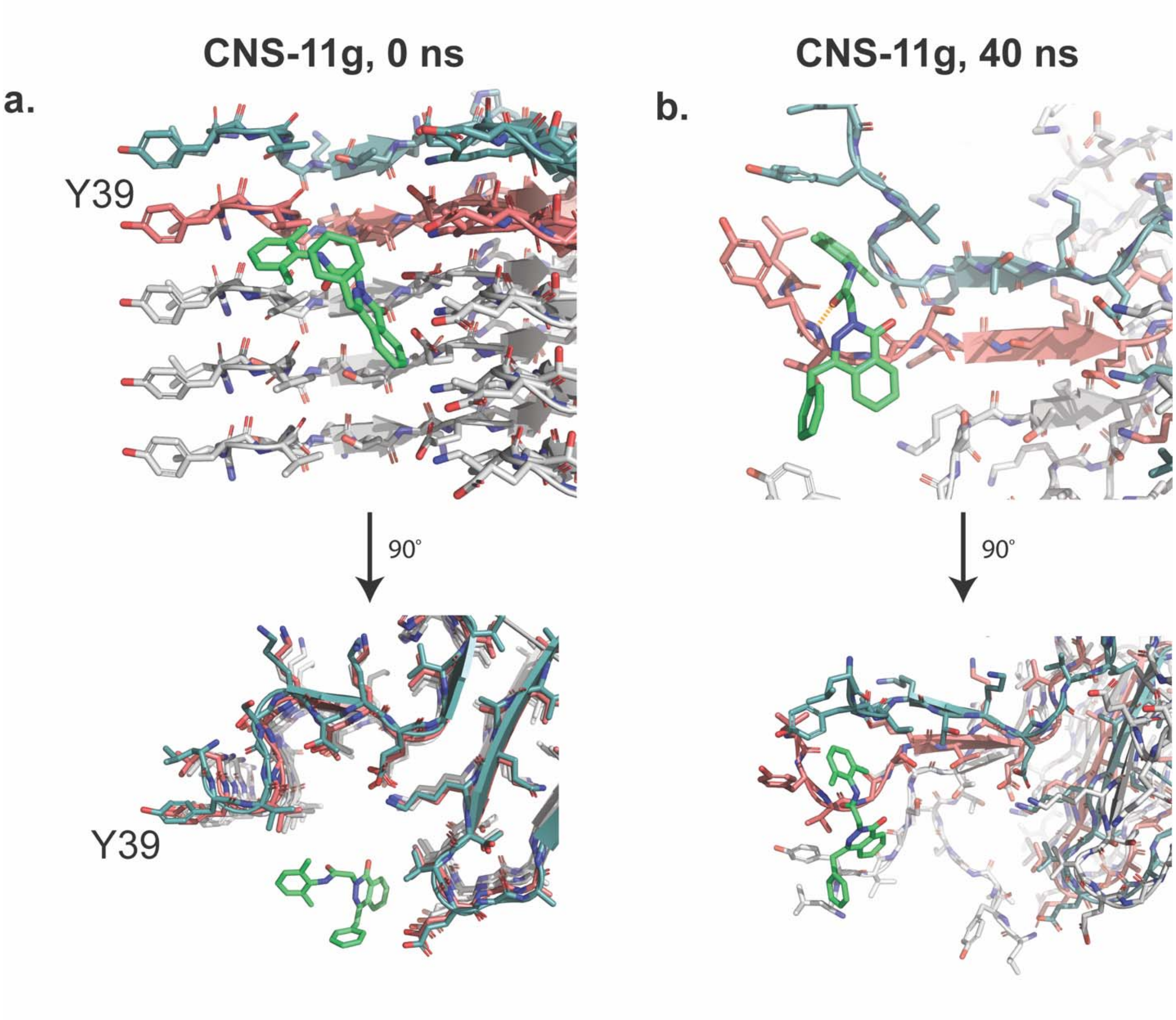

Supplemental Figure 6: Molecular dynamics simulation of CNS-11g with alpha-synuclein fibril. a. At the beginning of the simulation (0 ns), CNS-11g is docked to Site 2 of the recombinant alpha-synuclein fibril structure, near the N-terminus of the fibril core. The top layer of the fibril is colored blue/green, and the second layer is colored pink. Tyrosine 39 is highlighted. **b.** After 40 ns of simulation, significant separation of the strand layers at the N-terminus is observed. CNS-11g engages in hydrogen bonding with the amide backbone of alpha-synuclein (dashed yellow line), contributing to the destabilization of the fibril architecture.
